# Adaptation of a Sensory Reactivity Assessment for Use With Children in the Western Cape Province, South Africa

**DOI:** 10.1155/2024/5921153

**Published:** 2024-07-18

**Authors:** Ann Frances Watkyns, Lizahn Gracia Cloete, Linda Diane Parham

**Affiliations:** ^1^ Division of Occupational Therapy Department of Medicine and Health Sciences Stellenbosch University, Cape Town, South Africa; ^2^ Paediatrics Department of the School of Medicine University of New Mexico, Albuquerque, New Mexico, USA

**Keywords:** caregiver questionnaire, clinician-administered, performance-based, sensory modulation, sensory reactivity, test adaptation

## Abstract

**Background:** Sensory reactivity (SR) difficulties are characterised by problems regulating an individual's responses to sensory input such that it interferes with occupational performance in daily tasks. South African occupational therapists use assessments developed in the United States to identify SR difficulties in children. These have been found to be inappropriate for the South African context. This study reports on the methodology used to adapt an assessment for use in the Western Cape Province of South Africa.

**Method:** The SPM-2 Child and Preschool caregiver self-report questionnaires and seven tests of the performance-based Evaluation in Ayres Sensory Integration (EASI) that assess SR were identified for adaptation. A qualitative methodology was used to identify challenges using the assessment in a sample of the Western Cape population. Cognitive interviews were conducted with six community members from diverse socioeconomic groupings. Inductive analysis was used to identify and group the emerging themes. The test adaptation was conducted by expert occupational therapists based on these findings.

**Findings:** Challenges were grouped into two themes, namely, language challenges, of which there were three subcategories and inappropriate or threatening assessment tasks. Fifty-three changes were made to the assessment.

**Conclusion:** A detailed methodology was developed to adapt a SR assessment for use in the Western Cape Province. Challenges in using the assessment were elicited primarily from community members rather than professionals.

## 1. Introduction

Sensory reactivity (SR) difficulties are characterised by problems regulating or modulating the individual's responses to sensory input such that they interfere with occupational performance in daily tasks [1]. SR difficulties can present in children and adults. In children, SR difficulties can impact the childhood occupations of sleep, play, eating, social participation, concentration, and education and can affect mental health [2]. SR difficulties comprise sensory hyperreactivity and sensory hyporeactivity [3]. Sensory hyperreactivity refers to an exaggerated, frequently negative behavioural response to sensory input [2]. Sensory hyporeactivity denotes a reduced or absent response to sensory input [2].

SR is a component of the Ayres Sensory Integration® (ASI®) frame of reference developed by Dr. Ayres. Sensory integration as defined by Ayres is the organising and perceiving of sensory input which enables meaningful function and participation. ASI® currently comprises the four categories of SR, sensory perception, praxis, and ocular/postural/bilateral motor skills [2, 4]. This study focused on only one component of the ASI® assessment process, namely, the assessment of SR.

Assessment to identify SR difficulties has historically been done using a caregiver questionnaire [5–7]. The child's caregiver completes a Likert rating scale where they rate the frequency of behaviours that are commonly associated with SR difficulties. This method of evaluation has advantages and disadvantages. Advantages include that this method is cost-effective and quick to administer and provides information across a variety of contexts [1, 5, 8]. Disadvantages include the strong language base and subjective nature of the questionnaire, where the caregiver's emotions, culture, and parenting styles may influence their responses in the questionnaire [5, 8]. To address these disadvantages, the current best-practice recommendation is for a caregiver questionnaire to be used in conjunction with a clinician-administered, performance-based assessment [5, 7]. This approach allows for triangulation of results, thus enhancing the diagnostic accuracy in the identification of SR difficulties. There are however few performance-based assessments available, although some are currently in development or have been recently developed [4, 9–11].

Most SR assessments have been developed in the United States, published in English, and normed on the US population [12]. The two most frequently used assessments, both caregiver questionnaires, are the first and second editions of the Sensory Processing Measure (SPM) and the Sensory Profile [12]. Both questionnaires have been translated into several other languages [12–19].

Questionnaires used in different contexts can impact reliability and validity. In some countries, they were reported to be easy to administer with adequate reliability and validity [20]. However, many studies reported that reliability and validity of the questionnaires were affected by cultural and language differences, with items that were inappropriate for certain contexts requiring cultural adaptation [19, 21]. An example of a culturally inappropriate statement was an item in the Hong Kong translation of the SPM [17], which stated “Gags at the thought of an unappealing food, such as spinach.” In Hong Kong, spinach is not considered an unappealing food [17]. The researchers therefore removed this item from the Chinese translation [17]. Most adaptations of SR assessments have been done in the last 10 years [14, 15, 19, 22, 23], indicating the growing realisation of the importance of contextually appropriate assessments.

Only one translation and adaptation has been done on the African continent, in Ethiopia [19]. The SPM Preschool caregiver and teacher questionnaires were translated into Amharic, the most widely spoken language in Ethiopia, and were then culturally adapted [19]. The cultural adaptation involved replacing objects that were unfamiliar in the Ethiopian context with contextually appropriate, equivalent items. Examples of terms that were replaced were raincoat, pizza, and elevator [19].

Most adaptations reported in the literature have been conducted on caregiver questionnaires [16, 18, 19, 23]. Only one study could be found that described the adaptation of a performance-based assessment [21]. This was a translation and cultural adaptation of the Evaluation in Ayres Sensory Integration (EASI) for Spanish-speaking populations [21]. This may be due to two reasons. One reason is that questionnaires are more dependent on language than assessments administered by a clinician, with more challenges to their use in a different context. Second, most performance-based assessments are relatively new and may not yet have been adapted to diverse contexts [4, 9, 24].

Accuracy of the results of a questionnaire may also be influenced by cultural and language factors [25] and varying educational and reading levels [26] that impact the participants' understanding of the Likert scale. The words or numeric values used to define the scoring on the scale, the number of choices on the scale, the concept of ordering measurements on a continuum [25], and reading ability of the participants [26] were some of the factors that affected participants' responses.

Prior to test adaptation, information on the challenges identified in using the assessment is required. The most extensive feedback in test adaptation studies found in the literature was a study reporting on the translation and adaptation of the Short Sensory Profile into Malay for Malaysia [23]. In the study, feedback was elicited from 20 professionals, of whom 13 were occupational therapists. The remainder were language experts and one paediatrician [23]. In six additional studies, the number of panel members varied from 4 to 30 and included a varying mix of professionals and caregivers [13, 15–19]. These studies reflect an emphasis on eliciting feedback primarily from professionals.

The COnsensus-based Standards for the Selection of health status Measurement INstruments (COSMIN) researchers recommend that feedback to identify challenges in the use of an assessment be elicited primarily from patients [27]. Patients are considered to be experts on their condition, rather than the professionals, and their opinions therefore carry more weight than those of the professionals [27]. However, in the literature on adaptation of SR assessments, the term “expert” is frequently used to refer to professionals [15]. This change in emphasis recommended by COSMIN researchers corrects a historical imbalance inherent in the medical model, where the professional is considered the expert with all the necessary information on the patient's condition, and the power dynamic is weighted on the side of the professional [28].

One method for eliciting feedback from participants on assessments is cognitive interviewing. Cognitive interviewing is a semistructured, in-depth interview process that aims to understand the cognitive processes involved in and possible barriers to the completion of an assessment [29]. Types of questions asked include the following: What does the term mean to you? Was the rating scale easy or hard to understand? Can you repeat the statement in your own words? Would you like any question removed or changed? [29]

No SR assessment has been developed previously in South Africa, and there has been no adaptation conducted on a SR assessment to make it contextually appropriate for South Africa. South African occupational therapists have therefore used American assessments. These assessments were reported to be culturally inappropriate for the South African context [30–32]. The authors of these studies recommended research into the development of an appropriate SR assessment for South Africa [30–32].

South Africa is a very diverse country economically, culturally, educationally, and linguistically. Due to this highly heterogeneous population, it was unlikely that a single adaptation would be appropriate for all population groups. Therefore, the focus of this study was narrowed to the population of only one province, the Western Cape. This article describes the methodology used to adapt a SR assessment, comprising both a caregiver questionnaire and a performance-based assessment, suitable for use with children aged 3–12 years in the Western Cape Province of South Africa.

## 2. Methodology

The SR assessment most suitable for adaptation was identified using a rigorous systematic review process employing COSMIN methodology. Details of this process are provided elsewhere [33]. The SPM second edition (SPM-2) Preschool (ages 2–5 years) and Child (ages 5–12 years) caregiver questionnaires and the EASI (ages 3–12 years), a performance-based assessment, of which seven tests evaluate SR, were selected. Ethical approval to conduct the research was obtained from Stellenbosch University. The test adaptation was based on the methodology outlined by experts in the field [34, 35] and further informed by test adaptation studies conducted on SR assessments [16, 18, 19, 21, 23]. The five steps in the test adaptation process as adapted for this study are summarised in Table 1.

Permission was granted by the publishers of the SPM-2, Western Psychological Services, for adaptation of the six sensory subscales. The six sensory subscales were termed “vision” for visual input, “hearing” for auditory input, “touch” for tactile input, “taste and smell” as a combined subscale comprising gustatory and olfactory input, “body awareness” evaluating proprioceptive input, and the “balance and motion” subscale evaluating vestibular input [1]. Each subscale comprised 10 statements that the caregiver rated on a Likert scale according to the frequency of the behaviour described in the statement. The four rating options were *never*, *occasionally*, *frequently*, or *always* [1]. Two subscales evaluating praxis and social participation were omitted as they did not evaluate primary SR behaviours.

The EASI was developed as an open access assessment of ASI® [4]. The EASI comprised 20 tests that evaluate the core constructs of ASI® [4]. Seven of the EASI tests evaluate aspects of SR. These are vestibular hyperreactivity, auditory hyper- and hyporeactivity, tactile hyper- and hyporeactivity, oral hyper- and hyporeactivity, and olfactory hyperreactivity. The research version of the EASI test was used, as international normative data collection for the EASI was still underway when the present study was initiated.

The principal investigator (PI) invited members onto a technical committee according to predetermined selection criteria. The technical committee comprised six community members and four occupational therapists, in line with expert recommendations [34]. The purpose of the technical committee was to identify problematic aspects indicating the need for adaptation and to suggest alternatives. The occupational therapists were required to have a minimum of 10-year experience in the field of paediatric sensory integration and in measurement of the construct of SR.

Six community members were selected based on numerical guidelines in the literature [34]. They represented three different Western Cape communities, with two members from each community, reflecting the diversity of the Western Cape Province in regard to family income, culture, and language [34, 35]. Community members were recruited by the PI initially selecting three Cape Town suburbs representative of high, middle, and low socioeconomic communities according to the most recent census data. Ward councillors of these three communities were approached to provide the name of a community worker knowledgeable about their community members. The community worker then approached two community members who met the following study criteria. Parents or caregivers were required to be above the age of 18 years to provide legal consent as an adult and to have at least one child of 3–12 years in their care. Additional criteria were that parents or primary caregivers should not have an educational, medical, or clinical background, nor should they have had a child who had attended occupational therapy, to ensure that they were not familiar with the construct of SR, which may lead to biased responses. They needed to be proficient in English to a Grade 5 reading level to read the caregiver questionnaire in English [36]. The PI selected three communities in Cape Town that represented diverse socioeconomic (high-, middle-, and low-income levels), cultural, and language groupings (comprising the three official languages of the Western Cape Province with their related cultural characteristics). The selected occupational therapists and community members were requested to sign an informed consent form in their home language.

The occupational therapists were trained by the PI to enable them to fulfil their functions of identifying problematic aspects of the test and propose alternatives. Definitions of the construct of SR, hyporeactivity, and hyperreactivity as used in this study were presented and discussed. The term ASI® was clarified, as well as the positioning of SR in the ASI® framework. All four occupational therapists were familiar with the SPM or the SPM-2 questionnaires. Three of the four therapists were not familiar with the EASI, which requires postgraduate training to administer in a consistent, standardised manner. The PI produced a training video of the administration of the seven EASI tests for these three therapists.

Cognitive interviews were firstly conducted by the PI with the six community members to elicit their feedback on challenges using the tests. The interviews were done individually and face-to-face in the semistructured manner outlined in the cognitive interview guidelines [29, 37]. They were requested to choose a venue that was convenient for them, allowing for privacy to conduct the interview and in a space where they felt comfortable. The interviews were all video-recorded, with their permission. In addition, the PI wrote down all their verbal responses verbatim and checked their accuracy against the video recording.

The interviews all followed the same format, with the SPM-2 Preschool caregiver questionnaire presented first. Each community member was requested to read the questionnaire and think about how they would respond, bearing their child in mind. This was followed by the SPM-2 Child caregiver questionnaire and finally the seven tests of the EASI. As the EASI was a performance-based assessment, the test was demonstrated on the community members to allow them to fully experience the instructions and tasks involved in the administration. The PI elicited feedback on challenges and suggested adaptations throughout.

The video recordings of the six community member interviews were sent to the four occupational therapists. They watched the video recordings in their own time to familiarise themselves with the challenges identified by community members. They also read the SPM-2 questionnaires and viewed the EASI administration video to identify any additional challenges.

The challenges identified by the community members and the occupational therapists constituted the primary data. The thematic analysis was done manually, using inductive analysis, in which themes were not predetermined, but allowed to arise organically. The PI analysed the challenges to determine the reason why each highlighted issue was considered a problem. The reasons were then grouped thematically. Subthemes were grouped under each main theme according to the categories within the main theme. The identified problems were tabulated in a problem identification and rationale table that recorded the item number, the problems identified with that item, and the rationale behind the problem. This information guided the test adaptation process.

The test adaptation, based on community members' and occupational therapists' feedback, was conducted by the four occupational therapists and the PI. An alternative to each identified problem was agreed on by the group. The suggestions made by community members for ways to improve the assessment were considered and incorporated where possible. In instances where their suggestions were not used, the reason for this decision was noted. The adaptation agreed on by the group was reported in a problem identification and synthesis table.

The test adaptation was followed by a second round of cognitive interviewing with the same six community members a month after the first round of interviews. Feedback was obtained on the changes made by the occupational therapists. Further rounds of community member input were planned to allow for an iterative process until all challenges had been resolved. However, further community input was not needed, as the PI achieved redundancy in this second round and therefore no further interviewing was done. In addition to the adaptations made, gender-specific pronouns he/she were replaced with gender-neutral pronouns they/them/their.

A prefinal version of the SR assessment was created containing the agreed adaptations, as far as possible maintaining the original characteristics of the tests such as structure, format, materials, and scoring. The publishers granted approval to use the prefinal adapted version of the SPM-2 Preschool and Child caregiver questionnaires in a feasibility study. Details of the feasibility study are provided elsewhere [38].

## 3. Findings

Five of the community members were mothers and one was a caregiver. Four community members were between 40 and 42 years of age. The high-income level community members had higher educational qualifications than both the middle- and low-income level community members. Both low-income level community members spoke isiXhosa, and three of the remaining four community members spoke English. Demographic characteristics of the community members are provided in Table 2.

The demographics of the four occupational therapists represented a diverse group in terms of years of clinical experience, home language, and educational qualifications. One of the occupational therapists who requested participation fell short by 2 years on the experience criterion of 10 years. She was included due to her positive attributes of having a master's degree in the field and having been a reviewer for the systematic review conducted prior to the test adaptation and thus being familiar with the literature on SR. Detailed demographic information on the participating occupational therapists is provided in Table 3.

Cognitive interview feedback on the SPM-2 and the EASI indicated numerous challenges in using the assessments. These challenges were identified by community members and corroborated by the occupational therapists, who did not identify any additional challenges. Two themes emerged from the analysis of the cognitive interview primary data. Theme one represented challenges related to language. These all related to the caregiver questionnaires, as reading the questionnaire was a language-rich task. Theme two represented challenges related to inappropriate or threatening assessment tasks. These related to the performance-based assessment. These challenges will be discussed in more detail below, with selected quotes from community members presented in italics.

Most of the theme one challenges resulted from English being the community members' second or third language. The challenges fell into three subthemes, namely, Americanisms, contextually inappropriate terms, and language complexity and statements lacking clarity. *Americanisms* involved instances of American spelling and terminology. There were six instances where there was a difference between the American spelling of a word and the South African spelling, for example, “odor” is American and “odour” is South African. The different spelling caused confusion with some community members. There were six uses of American terminology that differed from terms used in South Africa, for example, the South African term “public toilets” is equivalent to the American term “public restrooms.” One test item contained two *contextually inappropriate terms*, with the use of the words lawnmower and air conditioner. Community Member 5: *Another one, ‘lawnmower'….I do not even understand what it means.*

Most language challenges were identified in the language subtheme *language complexity and statements lacking clarity*. Twenty-three words or phrases were not understood by some community members. Community Member 4: *I cannot mention the word right…reponising or rectonising?* [for recognising]. Community Member 5: *I do not understand what that means* [gags], *but when I see ‘vomit' after that, it is similar, so I can work it out.* Community Member 2 identified a statement which she felt lacked clarity, which is an aspect of this theme: *Both my kids dislike brushing teeth, but they aren't sensory sensitive. Maybe it should be reworded as ‘dislikes the feeling of brushing teeth'.*

The second theme related to *inappropriate or threatening assessment tasks* related to two of the performance-based EASI assessment tests. The tactile perceptions: Oral test required the child to put various shapes in their mouth and identify them by finding the matching shape on a printed card. Community Member 2: *That's quite challenging.…I'd be concerned whether there was stringent disinfecting. I wonder whether my kids would do this, and it's quite affronting*. In the SR test, one item required the child to sit on a chair and then be unexpectedly and rapidly tilted backwards by the tester. The other item required the child to stand on one chair and step onto another chair with a gap in between them. For both items, the child's emotional reaction and verbal responses were noted. Community Member 1: *That's pretty scary….and are kids OK with that, or do they get upset with you? That seems the most dangerous, but if it's a solid chair and it's just a little, then it's fine. I do not like that one, I think that's a little bit cruel. I do not like getting a fright, so for me, I would be upset by that.* Community Member 3: *They will be scared of that….it all depends how the height of the chair is…this height* [indicating the chair used by the PI to demonstrate the item] *there's no problem, that's fine, they will go with that.*

Eighteen items in the Preschool caregiver questionnaire, 22 items in the Child questionnaire, and 3 items in the performance-based assessment required adaptation. The challenges identified by community members were tabulated according to test items linked to the relevant themes and subthemes. They are presented in Table 4.

The suggested alternatives agreed on by the occupational therapists were then drawn up into a problem synthesis table stating the identified problems and the suggested alternatives, which are italics. This can be viewed in Table 5.

A second round of cognitive interviews with community members was conducted to obtain their feedback on the adaptations suggested by the occupational therapists. The adaptations were acceptable to all community members, with no further changes required. Their comments reflected this: Community Member 1: *Yes, that's perfect…ja* [yes], *I can see that it's like a little more simplified and a little more South African.* Community Member 2: *Yes, those all sound like good substitutes.* Community Member 3: *I'm happy with the changes.* Community Member 5: *Yes, it makes sense now.* Ten gender-specific words in the SPM-2 Preschool and Child questionnaires were replaced with gender-neutral wording as shown in Table 6, with the changes italics.

The incorporation of the adaptations of the SPM-2 Preschool and Child and EASI tests proposed in Tables 5 and 6 constituted the prefinal version of the SR assessment as adapted for the Western Cape Province of South Africa.

## 4. Discussion

This article reports on the methodology and adaptations made towards developing a valid and contextually appropriate SR assessment for the Western Cape Province of South Africa. The selection of both the SPM-2 Preschool and Child caregiver questionnaires and seven tests of the performance-based EASI aligned with recent best-practice recommendations that a subjective caregiver questionnaire and an objective performance-based assessment be used in combination when assessing a child for possible SR difficulties [39]. This adds richness to the assessment findings, facilitates triangulation of results from both sources, and allows for the development of a more holistic picture of the child's strengths and SR vulnerabilities [39].

The adaptations made to the SPM-2 caregiver questionnaires and the EASI were primarily based on the feedback obtained from cognitive interviewing of community members from diverse socioeconomic backgrounds. Feedback was also obtained from occupational therapists on the technical committee, but this was secondary to that of community members. The occupational therapists did not add any further information to what had already been provided by the community members. This aligned with the methodology recommended by Wild et al. [34] and the International Test Commission [35], who emphasised eliciting feedback from representative members of the target population rather than from professionals to guide the adaptation. Furthermore, the COSMIN guidelines for evaluating comprehensibility, acceptability, and relevance of an assessment recommend that this information be elicited from patients rather than professionals [27]. As patients were not relevant in this study, community representatives were the comparable nonprofessionals used to identify problematic items requiring adaptation. This methodology differed from the methodology used in SR test adaptation studies in the literature, which emphasised feedback from professionals to identify problematic items requiring adaptation [16, 18, 19, 21, 23]. The focus on obtaining feedback from community members was considered important in this study, as professionals could not effectively or objectively evaluate comprehensibility of an assessment due to their prior knowledge and familiarity in the field of SR.

Thematic analysis was used to categorise the data obtained from community members. Thematic analysis is a method used to analyse and interpret qualitative data whereby the data is examined to determine patterns and find themes [40], which was the type of analysis required in this study. Inductive thematic analysis allowed for the data to drive the analysis, with categories and themes arising organically from the primary data rather than being predetermined [40]. This technique enhances the validity of the findings and helps to prevent PI bias. If the themes had been preselected according to the PI's thoughts on the likely themes, some important issues may have been overlooked.

The challenges identified by community members indicated two themes. One theme related to language challenges when completing the caregiver questionnaire. Most of the challenges related to this theme. This was expected, given that the caregiver questionnaire had a strong language base and required a certain reading level in English to enable the caregiver to understand and complete it. This presented difficulties for community members whose home language was not English, many of whom resided in low socioeconomic areas. The public schools in these low socioeconomic areas frequently provide poor education. A recent study reported that 78% of Grade 4 learners were unable to read with understanding [41]. This poor reading ability is likely to persist into adulthood, impacting functional adult literacy levels, even though the caregivers met the Grade 5 reading level requirement specified in the selection criteria [36]. This resulted in difficulties reading and completing the caregiver questionnaire.

The theme related to language challenges had three subthemes, namely, Americanisms, contextually inappropriate terms, and language complexity or statements lacking clarity. The language subtheme related to Americanisms (American spelling or American terminology) appeared to have been adequately addressed in the adaptation. This was captured by one community member's statement expressing satisfaction that the American flavour had been replaced with a South African flavour: “*Yes, that's perfect…ja* [yes], *I can see that it's like.……a little more South African.*” This aided comprehension of the questionnaire and created a sense of ownership, which was likely to make caregivers more invested in completing the questionnaire accurately [27].

The language subtheme related to contextually inappropriate terms identified one item containing the words lawnmower and air conditioner. These challenges were reported by community members from low socioeconomic communities who noted that their homes did not have gardens and lawnmowers, with air conditioners also being outside their children's life experiences. These inappropriate terms impacted acceptability of the questionnaire, as the language was contextually and culturally inappropriate. Similar challenges have been reported in other studies on adaptation of caregiver questionnaires [14, 19]. Translation and adaptation of the Sensory Profile for Turkey found words such as vacuum cleaner and finger paint to be inappropriate, as these objects were not widely known [14]. Similarly, in the Ethiopian translation and adaptation of the SPM Preschool, words such as refrigerator, pizza, raincoat, and flushing toilet were replaced with more appropriate, widely used items [19]. When a questionnaire contains items that are inappropriate for the participants, there may be a disconnect and sense of alienation, which affects motivation to complete the questionnaire and can impact validity [27].

The language subtheme of challenges related to complexity of the language or statements lacking clarity impacted comprehensibility of the questionnaire. Most challenges fell into this category. Comprehensibility was also the most common aspect identified in the literature where challenges were identified [13, 15, 17]. Afrikaans- and isiXhosa-speaking community members at times struggled to understand more complex English words such as “distracted, distressed, recognising, and wincing.” They were therefore disadvantaged in completing the questionnaire, which may have affected the accuracy of their responses. Translation into the caregivers' home language would likely address most of these challenges and greatly facilitate understanding and more accurate completion of the questionnaire, with positive effects on validity of the assessment.

Another aspect of this subtheme concerned statements lacking clarity, which also impacted comprehensibility. This challenge was identified infrequently and reported across socioeconomic and language groupings. An example was SPM-2 Child Item 27 “dislikes brushing their teeth,” to which a caregiver responded *Both my kids dislike brushing teeth, but they aren't sensory sensitive. Maybe it should be reworded as ‘dislikes the feeling of brushing teeth'.*

The second theme comprised three inappropriate or threatening assessment tasks relating to two EASI tests, the tactile perception: oral and the SR tests. The tactile perception: Oral test requires the child to identify various shapes placed in their mouth. The first round of cognitive interviews took place during the COVID pandemic, and COVID regulations required frequent sanitising and the wearing of masks. Community members may have had greater sensitivity to the hygiene implications of placing objects in their mouths and valid concerns regarding adequate sanitising. These factors may have contributed to their negative responses.

Other items deemed inappropriate or threatening were two SR test items involving testing the child's balance. The items require the clinician to place significant demands on the child's balance. The two community members who commented on this item used similar words in their feedback: *They will be scared of that*, and *That's pretty scary*. Items such as these may raise ethical, psychological, legal, or social concerns because they are likely to induce anxiety and fear for some children [42].

The test adaptation methodology described in this article allowed for an iterative process between community members and occupational therapists. This would have been repeated until all identified challenges were resolved. However, this was not necessary, as all the community members' concerns were addressed in the first round of test adaptations.

This study had two limitations. The power relationship between the PI and community members during cognitive interviews could potentially be weighted in the PI's favour due to the PI being the study leader, being knowledgeable in the field, and many community members struggling to read the English caregiver questionnaire, as English was their second or third language. Their sense of competence and confidence in the interview situation may therefore have been affected. The PI addressed the potential imbalance of this power relationship by emphasising that the community members played an important role in the research process and provided valuable information. However, despite the efforts made by the PI to equalise the power relationship, some community members may have still been affected by what they perceived as an imbalance in the power relationship.

The second limitation was the use of the original English version of the caregiver questionnaires for the test adaptation. Numerous comprehensibility challenges were identified by Afrikaans- and isiXhosa-speaking community members. Many of these challenges would not have been identified had the questionnaires been translated into Afrikaans and isiXhosa.

Recommendations for further research include a larger study to evaluate psychometric properties and to develop norms for the Western Cape population. Translation into the other two official languages of the Western Cape Province, isiXhosa and Afrikaans, is also recommended. When caregivers can read the questionnaire in their home language, this contributes to an equalising of the power relationship and eliminates many comprehension difficulties identified in this study.

## 5. Conclusion

There was no SR assessment, new or adapted, appropriate for use in South Africa, prior to this study. South African occupational therapists experienced challenges using assessments developed in other countries, to assess SR difficulties [30–32]. This study describes a systematic, detailed methodology for performing a test adaptation for a different population and a new context. The adaptation was based primarily on feedback from community representatives in the target population, whereas most adaptations of SR assessments relied either solely or for the most part on feedback from a variety of professionals such as occupational therapists, psychologists, and language experts [16, 18, 19, 21, 23]. This community-based adaptation provides a methodology that can be used in future test adaptations. It was unique in that it involved an adaptation of both a caregiver questionnaire and a performance-based assessment. It was also the first adaptation of a SR assessment to be conducted in South Africa and only the second adaptation of an SR assessment reported on in Africa [19]. Findings from this study highlight the importance of adapting a test when used with a population or within a context that differs significantly from those in which the test was developed.

## Figures and Tables

**Table 1:** Five steps in test adaptation (adapted) [34–36].

**Step**	**Method**	**Description**	**Rationale**
1	Preparation	Obtain permission from publisher to adapt the test	To respect the copyright
		Enquire about any methodology stipulated by publisher for test adaptation	The publisher may have certain stipulations on test adaptation which may impact on adaptations planned
		Develop clear definitions of the construct to be measured and other relevant concepts and terms involved	All professionals involved in the adaptation process need to have the same definitions of constructs and other concepts involved in the study To avoid confusion or ambiguities in terminology
		Select technical committee comprising four expert occupational therapists and six community members representative of the target population	Having additional experts on the committee assists in minimising bias in data collection and analysis and allows for a range of inputs from a diverse group Input from a sample of the target population is necessary to provide feedback on comprehensibility and appropriateness of the test

2	Cognitive interviewing, Round 1	With the technical committee members, firstly community members, then occupational therapists	To identify potential problems with the recommended assessment regarding comprehensibility, relevance, acceptability, and comprehensiveness

3	Cultural/contextual language adaptation	Same language translation for a different context and population to the original	To prevent ambiguity, misunderstanding and misinterpretation To ensure cultural and contextual appropriateness To ensure language comprehensibility

4	Cognitive interviewing, Round 2	With the technical committee members	To check for comprehensibility, relevance, acceptability, and comprehensiveness
	Revision	Revision of adapted test as needed	To correct any further problems identified
	These two steps continue in an iterative process until all challenges have been adequately addressed		

5	Proofreading	Final quality control	To check for spelling, grammatical, or other errors To ensure a final version that is free of errors To create a version that is visually similar to the original

**Table 2:** Demographic characteristics of community members on technical committee.

	**Role**	**Age in years**	**Educational level**	**Community income level**	**Home language**
Community Member 1	Mother	42	Diploma^a^	High	English
Community Member 2	Mother	41	Honours degree	High	English
Community Member 3	Mother	40	Grade 9	Middle	Afrikaans
Community Member 4	Caregiver	23	Grade 10	Middle	English
Community Member 5	Mother	40	Grade 11	Low	isiXhosa
Community Member 6	Mother	21	Grade 12	Low	isiXhosa

^a^In South Africa, a diploma is a postschool educational qualification of usually 2 years and is positioned below an undergraduate degree.

**Table 3:** Demographic characteristics of occupational therapists on technical committee.

	**Educational level**	**Experience in paediatric SI (years)**	**Public/private work setting**	**Home language**	**Familiar with the EASI**	**Familiar with the SPM/SPM-2**
OT 1	Master's	8	Public	English	Yes	Yes
OT 2	Undergraduate degree	36	Private	English	No	Yes
OT 3	PhD	16	Private	Afrikaans	No	Yes
OT 4	Undergraduate degree	12	Private	English	No	Yes

Abbreviations: EASI = Evaluation in Ayres Sensory Integration; OT = occupational therapist; SI = sensory integration; SPM-2 = Sensory Processing Measure second edition.

**Table 4:** Challenges identified with original wording or task and their rationale.

**Item no.**	**Problem with original wording or task**	**Rationale**
*SPM-2 Preschool*		
Instructions	Behaviour	American spelling
	Frequently = much of the time	American terminology
	Wincing	Language complexity or statement lacking clarity
	Distressed	Language complexity or statement lacking clarity
9	Store	American terminology
12	Responds to loud noises by running away	Language complexity or statement lacking clarity
13	Is distracted or bothered by background noises that others ignore, such as a lawnmower outside or an air conditioner	Contextually inappropriate term
19	Has difficulty following verbal directions	Language complexity or statement lacking clarity
20	Has difficulty determining the location of sounds or voices	Language complexity or statement lacking clarity
28	Saliva	Language complexity or statement lacking clarity
32	Fails to distinguish or indicate preference among flavors	American spelling; language complexity or statement lacking clarity
36	Odors	American spelling
39	Seeks out certain scents or odors	Language complexity or statement lacking clarity
40	Restrooms	American terminology
44	Pet animals	American terminology
54	Fails to catch himself or herself when falling	Language complexity or statement lacking clarity
57	Rocks, sways, or squirms when seated	Language complexity or statement lacking clarity
78	Transitions smoothly to new activities	Language complexity or statement lacking clarity

*SPM-2 Child*		
Instructions	As for preschool form	
3	Colorful	American spelling
5	Has difficulty recognizing how objects are similar or different	Language complexity or statement lacking clarity
6	Dislikes certain types of lighting, such as strobe lights, flickering lights, or fluorescent lights	Language complexity or statement lacking clarity
8	Stores	American terminology
9	Is distracted by visible objects or people	Language complexity or statement lacking clarity
13	Fails to notice sounds that others notice	Language complexity or statement lacking clarity
15	Is frightened of sounds that do not usually distress others	Language complexity or statement lacking clarity
27	Dislikes brushing his or her teeth	Language complexity or statement lacking clarity
29	Saliva	Language complexity or statement lacking clarity
35	Odors	American spelling
39	Restrooms	American terminology
40	Fails to distinguish or indicate preference among flavors	American spelling; language complexity or statement lacking clarity
44	Jumps a lot	Language complexity or statement lacking clarity
48	Deliberately bangs head into objects or people	Language complexity or statement lacking clarity
55	Dislikes tilting the head backwards	Language complexity or statement lacking clarity
58	Squirms	Language complexity or statement lacking clarity
69	Takes excessive time to complete routine tasks	Language complexity or statement lacking clarity
70	Has difficulty generating ideas for what to make	Language complexity or statement lacking clarity

*EASI*		
TP: oral	All items	Inappropriate or threatening assessment task
SR test, Item 15	Tipping chair back suddenly	Inappropriate or threatening assessment task
SR test, Item 17	Climbing from one chair to another	Inappropriate or threatening assessment task

Abbreviations: EASI = Evaluation in Ayres Sensory Integration; SPM-2 = Sensory Processing Measure second edition; SR = sensory reactivity; TP = tactile perception.

**Table 5:** Challenges identified with original wording or task and suggested alternatives.

**Item no.**	**Original wording or task**	**Suggested alternative**
*SPM-2 Preschool*		
Instructions	Behavior	*Behaviou*r
	Frequently = much of the time	Frequently = *most of the time*
	Wincing	Delete
	Distressed	*Becomes upset*
9	Stores	*Shops*
12	Responds to loud noises by running away…	Responds to loud noises, *such as a door banging*, by running away…
13	Is distracted or bothered by background noises that others ignore, such as a lawnmower outside or an air conditioner	Is distracted or bothered by background noises that others ignore, *such as a radio or TV on or music playing outside*
19	Has difficulty following verbal directions	Has difficulty following *simple* verbal *instructions*
20	Has difficulty determining the location of sounds or voices	Has difficulty *knowing where* sounds or voices *are coming from*
28	Saliva	*Spit*
32	Fails to distinguish or indicate preference among flavors	Fails to distinguish or indicate preference among *flavours such as sweet/sour/bitter*
36	Odors	*Odours*
39	Seeks out certain scents or odors	Seeks out certain *smells*
40	Restrooms	*Toilets*
44	Pet animals with too much force	*Pat* animals with too much force
54	Fails to catch himself or herself when falling	*Does not stop* himself or herself *from falling*
57	Rocks, sways, or squirms when seated	Delete sways; replace squirms with wriggles Rocks *or wriggles* when seated
78	Transitions smoothly to new activities	*Moves easily from one activity to another*

*SPM-2 Child*		
Instructions	As for preschool	
3	Colorful	*Colourful*
5	Has difficulty recognizing how objects are similar or different	Has difficulty *noticing* how objects are similar or different
6	Dislikes certain types of lighting, such as strobe lights, flickering lights, or fluorescent lights	Dislikes certain types of lighting, such as *sunlight*, flickering lights, or *bright lights*
8	Stores	*Shops*
9	Is distracted by visible objects or people	Is distracted by *looking at* objects or people *around him or her*
13	Fails to notice sounds that others notice	Fails to notice sounds that others notice, *such as ignores an instruction*
15	Is frightened of sounds that do not usually distress others	Is frightened of sounds that do not usually *upset* others, *such as a siren or a door banging*
27	Dislikes brushing his or her teeth	Dislikes *the feeling of* brushing his or her teeth
29	Saliva	*Spit*
35	Odors	*Odours*
39	Restrooms	Toilets
40	Fails to distinguish or indicate preference among flavors	Fails to distinguish or indicate preference among *flavours such as sweet/sour/bitter*
44	Jumps a lot	Jumps a lot so that it interferes with his or her daily activities
48	Deliberately bangs head into objects or people	Bangs his head into objects or people on purpose
55	Dislikes tilting the head backwards	Dislikes tipping the head backwards
58	Squirms	Wriggles
69	Takes excessive time to complete routine tasks	Takes too much time to complete routine tasks
70	Has difficulty generating ideas for what to make	Has difficulty thinking of ideas for what to make

*EASI*		
TP: oral	All items	Ensure sanitising procedures as in test instructions
SR test, Item 15	Tipping chair back suddenly	After asking the child for their response to the stimulus, say: *I will not do that again*
SR test, Item 17	Climbing from one chair to another	If the child resists or refuses to cooperate, this will be noted and the child will not be forced to do the item

Abbreviations: EASI = Evaluation in Ayres Sensory Integration; SPM-2 = Sensory Processing Measure second edition; SR = sensory reactivity; TP = tactile perception.

**Table 6:** Gender-neutral wording to replace gender-specific wording.

**Test and item number**	**Adapted gender-specific wording**	**Gender-neutral wording**
SPM-2 Preschool Item 7	Looks at objects out of the corner of his or her eye	Looks at objects out of the corner of *their* eye
23	Is upset by having his or her hair cut or brushed	Is upset by having *their* hair cut or brushed
54	Does not stop himself or herself from falling	Does not stop *themselves* from falling
SPM-2 Child Item 4	Has trouble following moving objects with his or her eyes	Has trouble following moving objects with *their* eyes
7	Looks at objects out of the corner of his or her eye	Looks at objects out of the corner of *their* eye
9	Is distracted by looking at objects or people around him or her	Is distracted by looking at objects or people around *them*
23	Becomes upset by having his or her fingernails or toenails cut	Becomes upset by having *their* fingernails or toenails cut
24	Seems bothered when someone touches his or her face	Seems bothered when someone touches *their* face
27	Dislikes brushing his or her teeth	Dislikes the feeling of brushing *their* teeth
44	Jumps a lot so that it interferes with his or her daily activities	Jumps a lot so that it interferes with *their* daily activities

Abbreviation: SPM-2 = Sensory Processing Measure second edition.

## Data Availability

The data is available from the first author on request.
